# The Role of Nuclear β-Catenin Accumulation in the Twist2-Induced Ovarian Cancer EMT

**DOI:** 10.1371/journal.pone.0078200

**Published:** 2013-11-11

**Authors:** Yubin Mao, Jinfei Xu, Zhihan Li, Nini Zhang, Hao Yin, Zuguo Liu

**Affiliations:** 1 Department of Pathophysiology in Basic Science, Medical College of Xiamen University, Xiamen, Fujian, China; 2 Eye Institute and Xiamen Eye Center, Medical College of Xiamen University, Xiamen, Fujian, China; Martin-Luther-University Halle, Germany

## Abstract

**Background:**

Twist2 has been shown to promote human tumor invasion as in breast cancer and cervical cancer. However, whether Twist2 promotes human ovarian cancer progression remains to be elucidated. Here, we investigate the role of Twist2 in ovarian cancer invasion and metastasis as well as the underlying molecular mechanisms.

**Methods:**

Twist2 expression was detected by Immunohistochemistry (IHC) on tissue microarray of human ovarian cancers with scoring procedure according to the staining intensity and pattern. Twist2 gene was stably introduced into SKOV-3 ovarian cancer cells to examine the changes of cellular morphology, motility, invasiveness, and EMT molecular markers.

**Results:**

Twist2 expression is significantly increased in ovarian cancers along with the FIGO disease stage, indicating that Twist2 may be associated with ovarian cancer metastasis. Overexpression of Twist2 induced the EMT phenotype including downregulation of E-cadherin, and upregulation of N-cadherin and β-catenin in human ovarian cancer cells, suggesting that Twist2 might promote β-catenin release from the E-cadherin/β-catenin complex through inhibition of E-cadherin. Thus, β-catenin degradation was inhibited due to inhibition of APC, and the Wnt/β-catenin pathway was then activated by nuclear β-catenin accumulation, which may activate transcription of downstream target genes to promote tumor invasion and metastasis. Collectively, these data indicated that β-catenin is involved in Twist2-induced EMT in ovarian cancer.

**Conclusion:**

Our data indicates that upregulation of Twist2 is correlated with the FIGO stage in human ovarian cancers. In this report, we demonstrated that nuclear β-catenin is accumulated in Twist2-induced EMT cells to facilitates ovarian cancer invasion and metastasis.

## Introduction

Despite recent advance in the understanding of ovarian cancer development and progression, ovarian cancer remains has the highest associated mortality rate of gynecologic malignancy in mostly western countries due to the advanced stage of disease at diagnosis (stages III–IV). The vast majority of women are diagnosed with disseminated intraperitoneal carcinomatosis [1], [2]. Recent literature suggests that the epithelial to mesenchymal transition (EMT) plays a critical role in the progression of ovarian carcinomas [3], [4]. EMT is defined by a complex molecular and cellular program by which epithelial cells lose their differentiated characteristics such as cell-cell adhesion, apical-basal polarity, lack of cell motility and gain of mesenchymal features including motility, invasiveness and increased resistance to apoptosis [5]. The similar cellular remodeling and signaling networks appear to be active during cancer metastasis [6], [7]. It has been suggested that primary EOC may undergo an EMT process during local invasion in the peritoneum and retain mesenchymal features in advanced tumors.

Transcription factors of the Twist family (Twist1, Twist2), snail family (SNAI1/snail, SNAI2/slug), as well as ZEB family (ZEB1, ZEB2), control the EMT process in cancer [5], [8], [9]. Twist2 has been shown to promote tumor progression through EMT in breast cancer [10]. Twist2 is a potential prognostic marker for cervical cancer [11], adenoid cystic carcinoma [12], and tongue squamous cell carcinoma [13]. Twist2 is also correlated with poor differentiation grade and short survival in head and neck squamous cell carcinomas [14]. However, whether Twist2 promotes human ovarian cancer progression remains poorly understood. Here, we investigate the role of Twist2 in ovarian cancer progression and the potential molecular mechanisms underlying the Twist2-induced cancer invasion and metastasis. We showed Twist2 is highly expressed in ovarian cancer tissues. Ectopic expression of Twist2 in ovarian cancer cells confers an EMT phenotype. Furthermore, β-catenin activation may participate in these Twist2 induced EMT properties.

## Results

### 1. Increased Twist2 expression was correlated with FIGO stage in primary ovarian cancer

**Figure 1:**
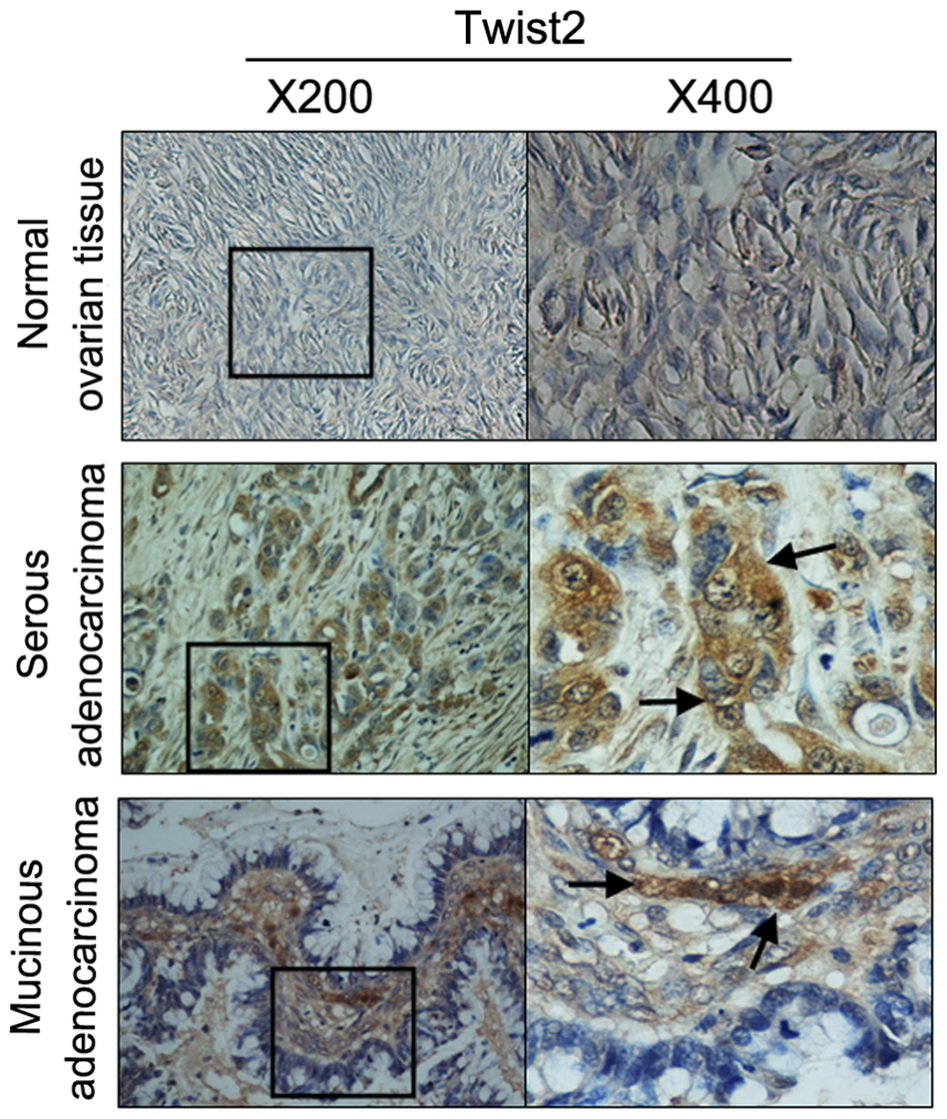
Immunohistochemical (IHC) staining of Twist2 expression on paraffin-embedded ovarian carcinomas tissue array sections. Tissue sections of ovarian carcinomas and normal ovaries were analyzed by IHC staining with a specific monoclonal antibody against human Twist2. The positive staining of Twist2 was shown in brown color. The sections were counterstained with hemotoxylin to show nuclei. Representative images of Twist2 staining in paired normal ovary and epithelial ovarian carcinomas are shown. No expression of Twist2 could been seen in normal adult ovary tissues, while positive expressions of Twist2 were mainly localized in cytoplasm or nucleus of the serous adenocarcinoma and the mucinous adenocarcinoma ovarian tumor cells. Magnitude ×200, ×400.

IHC analyses indicated the presence of high levels of Twist2 in cancer cells of primary ovarian tumors on tissue array (Figure 1). Twist2 expression was significantly increased in ovarian cancer (70.24%, 59 in 84 cases) relative to normal ovarian tissue (P<0.05, Figure 1, Table 1). The positive staining of Twist2 was detected both in nucleus and cytoplasm.

**Table 1:** Clinical pathological characteristics of Twist2-associated ovary cancer.

	Cases	Twist2				
		^_^	+	++	*X2*	*P*
Tissue type					12.16	<0.05
Normal ovarian tissues	10	9	1	0		
Malignant tumor	84	25	37	22		
Tumor histological type					4.01	>0.05
Serous	69	21	29	19		
Mucinous	8	3	4	1		
Endometrioid	2	1	1	0		
Clear cell	5	0	3	2		
FIGO stage					13.01	<0.05
I	44	14	20	10		
II	13	1	8	4		
III	14	8	5	1		
IV	13	2	4	7		

According to histological type classification, strong expression of Twist2 was found in 19 of 69 serous carcinomas (27.54%), 1 of 8 mucinous carcinomas (12.5%), none of 4 endometriod carcinomas (0%), and 2 of 5 Clear cell carcinomas (40%). No significant difference could be detected in different tumor histological type (P>0.05). The expression of Twist2 was increased along with the FIGO stage (P<0.05), exemplified as following: 22.73% at stage I (10 in 44 cases), while 53.85% at stage IV (7 in 13 cases). Overall, at the stage I, 31.82% (14/44) cases showed Twist2 negative expression, 45.45% (20/44) cases showed weak expression, and 22.73% (10/44) cases displayed strong expression; at the stage II, Twist2 negative 7.69% (1/13); weak expression: 61.54% (8/13), and strong positive: 30.77% (4/13); at the stage III, Twist2 negative expression: 57.14% (8/14), weak expression: 35.71% (5/14), and strong expression: 7.14% (1/14); at the stage IV, negative expression: 15.38% (2/13), weak expression: 30.77% (4/13), strong expression: 53.85% (7/13).

These data demonstrated that Twist2 protein is significantly increased in ovarian cancer tissues and the high level of Twist2 was correlated with FIGO disease stage, suggesting that Twist2 can be used as an indicator of high grade of malignancy and poor prognosis in human ovarian cancers. However there is no correlation between Twist2 expression and the tumor histological type.

### 2. Twist2 expression affected cell morphology but not the cell proliferation of SKOV-3 cells *in vitro*

**Figure 2:**
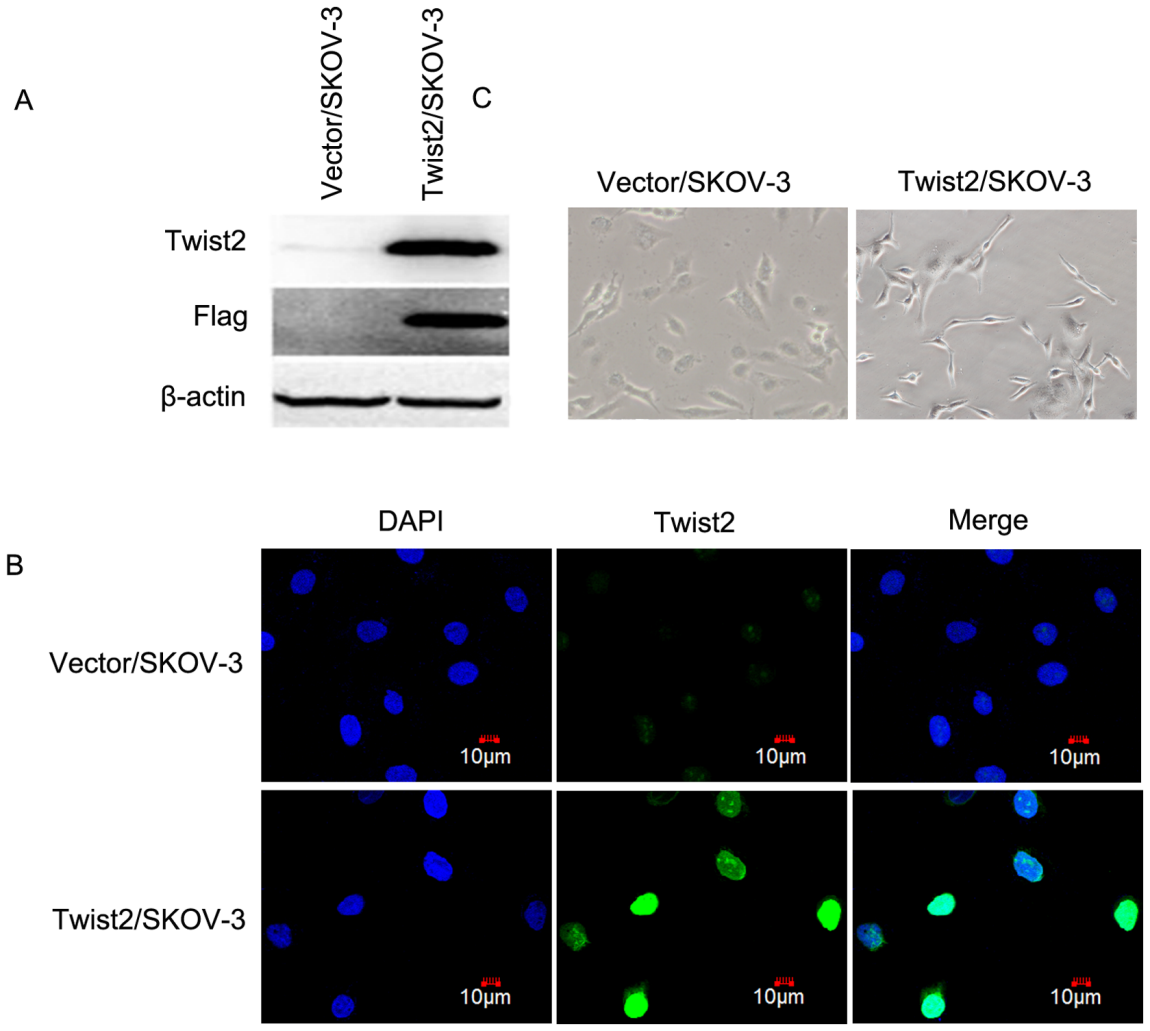
Generation of Twist2-overexpression stable ovarian cancer cells and cell morphological observation. **A.** Ectopic expression of the Flag-taggedTwist2 in SKOV-3 (Twist2/SKOV-3) ovarian cancer cells were verified by Western blot. Twist2/SKOV-3 cells expressed both Twist2 and flag-tag compared with vector control cells. **B.** Twist2 staining was observed using the laser scanning confocal microscopy (Olympus) in SKOV-3 cells. Nuclei were counterstained with DAPI (in blue). Immunofluorescent staining of Twist2 in Twist2/SKOV-3 cells showing cells with Twist2 (in green) in cell nuclei compared with the Vector control SKOV-3 cells. The cells were from the stably transfected samples. **C.** Cell morphological shapes were observed using phase microscopy (Olympus). Twist2/SKOV-3 cells took on the fibroblast-like morphological shape, while the Vector/SKOV-3 control cells appeared epithelial cell shape.

To verify whether Twist2 is a crucial mediator of ovarian cancer, progression, we established cell lines stably overexpressing Twist2 (Fig. 2A). We then analyzed Twist2/SKOV-3 cells and their parental cells through cell morphological observation, flow cytometry (FCM), and MTT assay.

In the initial characterization of these cells, we observed a distinct morphological change in the cells overexpressing Twist2 (Fig. 2B). The SKOV-3 cells expressing Twist2 underwent a phenotypic change and appear fibroblast-like mesenchymal morphological shape, while the control SKOV-3 cells (Vector/SKOV-3) appear planar epithelial cell morphological shape (Fig. 2B). Immuno-fluorescent staining showed that Twist2 is localized in nuclei, which was consistent with the fact that Twist2 is a transcription factor (Fig. 2C).

**Figure 3:**
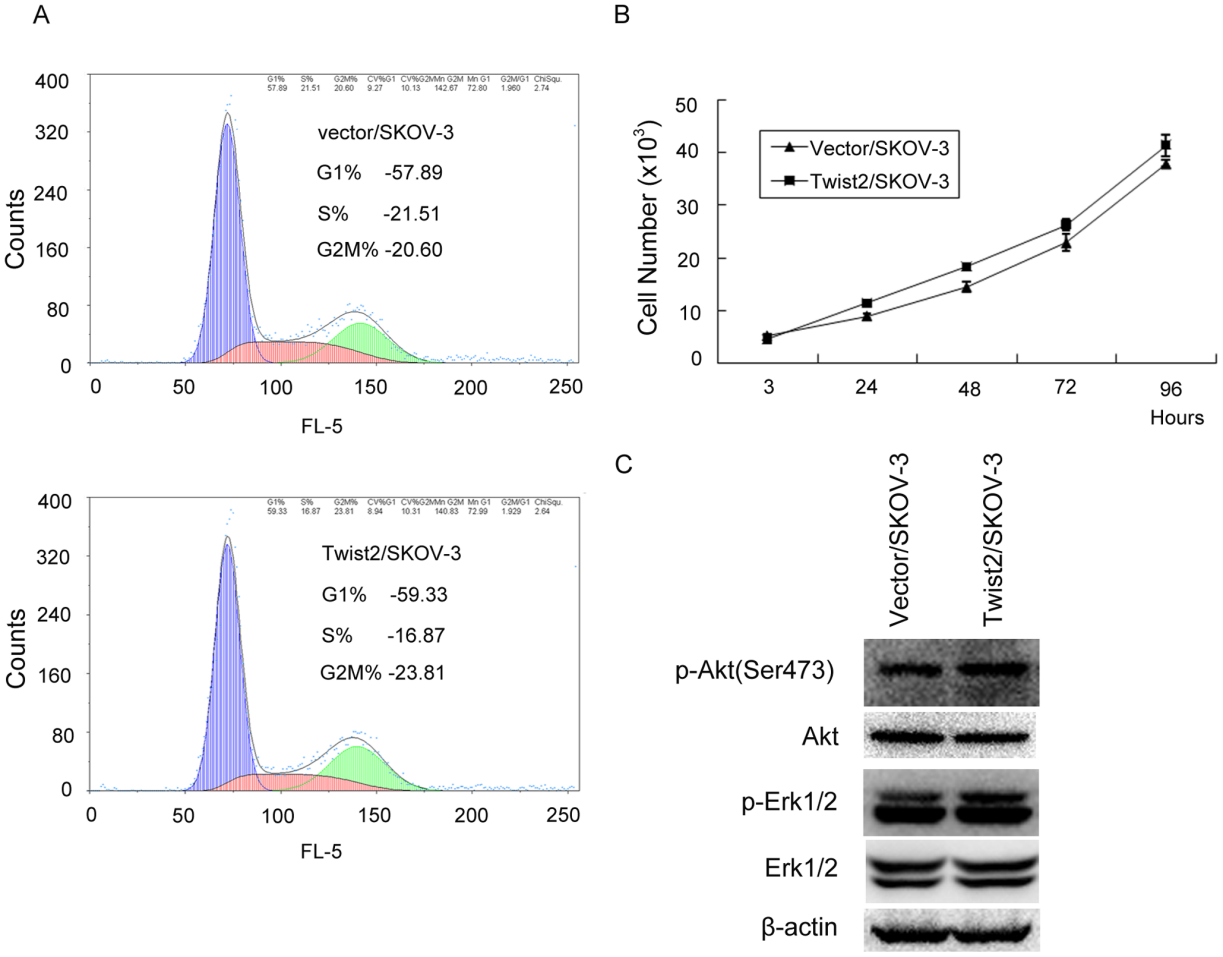
The proliferation influence of SKOV-3 cells by Twist2. **A.** Flow cytometry analysis showed no difference of the cell cycle distribution between SKOV-3 cancer cells with or without Twist2 ectopic expression. **B.** The proliferation rate of the transfected cells was detected and compared with that of the vector control through viable cell counts using trypan-blue staining. Triplicate assays were performed in each group of cells. Data were expressed as mean ± SD. No obvious influence of Twist2 was observed on cell growth rate. **C.** Expressions of Akt, Erk1/2, and their phosphoralation forms indicated Twist2 had no effects on proliferation by Western blot. Compared with Vector/SKOV-3 cells, no obvious changes of Akt, Erk1/2, and their phosphorylation forms were found in Twist2/SKOV-3 cells.

We then evaluated the proliferation of SKOV-3 cells expressing Twist2through analyses of cell cycle distribution and cell growth rate. The results showed no obvious effect of Twist2 expression on cell proliferation (Fig. 3A, 3B). As Akt and Erk1/2 are important indicators involved in cell proliferation, the expressions of these proteins and their phosphorylation were examined by Western blot analysis. Consistently, we did not detect changes in Akt, Erk1/2, and their phosphorylation forms in Twist2/SKOV-3 cells realtive to the Vector/SKOV-3 control cells (Fig. 3C).

### 3. Twist2 Expression Enhanced Migration and Invasion of SKOV-3 Ovarian Cancer Cells

**Figure 4:**
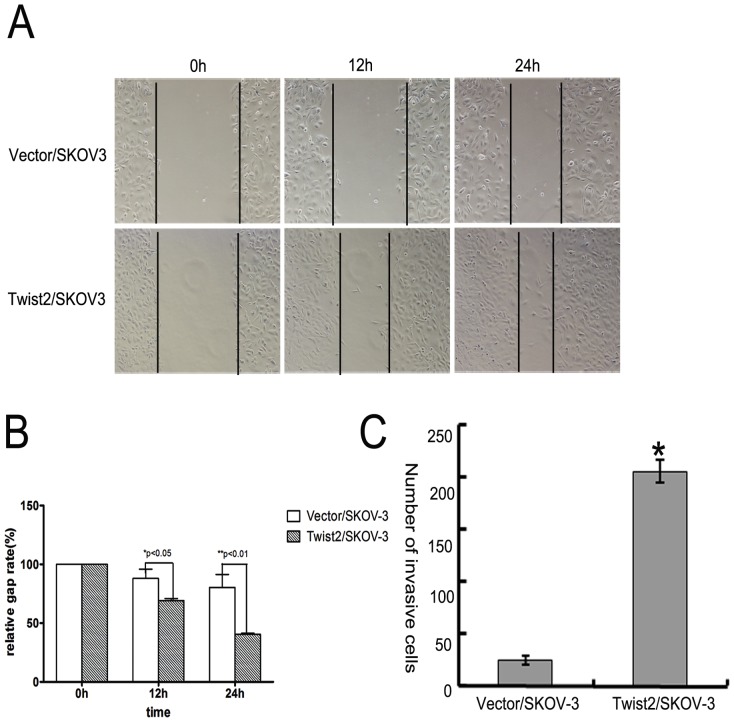
Overexpression of Twist2 in epithelial ovarian carcinoma cells increased cell migration, invasive potential *in vitro*. **A.** In vitro wound healing assay of SKOV-3 cancer cells with or without Twist2 ectopic expression. Representative images of cells at “healing” sites at 12 and 24 hours after scraping are shown increased cell migration in Twist2/SKOV-3 cells. **B.** The analyzed migration results by the one way ANNOVA test (Graphpad Prism5 software). A P-value of 0.05 was considered statistically significant. The Twist2-expressing SKOV-3 cells migrated faster than the vector-transfected control cells. **C.** In vitro matrigel invasion assay of SKOV-3 cells with or without ectopic expression of Twist2. Quantification indicated that Twist2-expressing cells are 8–9 folds more invasive than the vector control cells in the in vitro matrigel invasion assay. *, *p*<0.005.

Twist2 has been shown to be an important inducer of EMT in breast and cervical cancer. EMT process promotes epithelial cells to appear fibroblast shape, characterized by increased motility and invasiveness [15]. To determine whether Twist2/SKOV-3 cells have acquired the increased ability to migrate, we performed a wound healing assay in which the motility of cells located at the edge of the wound was evaluated on the basis of their ability to colonise the wounded area. In the presence of serum (10% FBS), wound repair was observed faster in the Twist2/SKOV-3 cells than control cells group at 24 h (Fig. 4A). As there was no increase in proliferation induced by Twist2 (Fig. 3C), the Twist2/SKOV-3 cells might have the greater cell migration potential *in vitro* in “wound healing” assay (Fig. 4A, 4B). These results were confirmed by a transwell chamber assay containing an extracellular matrix layer (matrigel). As shown in Figure 4C, the number of Twist2/SKOV-3 cells migrated through the pores is more than the control cells did.

### 4. Nuclear β-catenin was accumulated in ovarian carcinoma cells when Twist2 induced EMT phenotype

When Twist2 over-expresses in the ovarian cancer cells, the cancer cells morphology was transformed into fibroblast-like shape along with increased motility and invasiveness, while no change was found in cell proliferation. Our data indicated that forced expression of Twist2 might push these EOCs to undergo an epithelial-mesenchymal transition.

**Figure 5:**
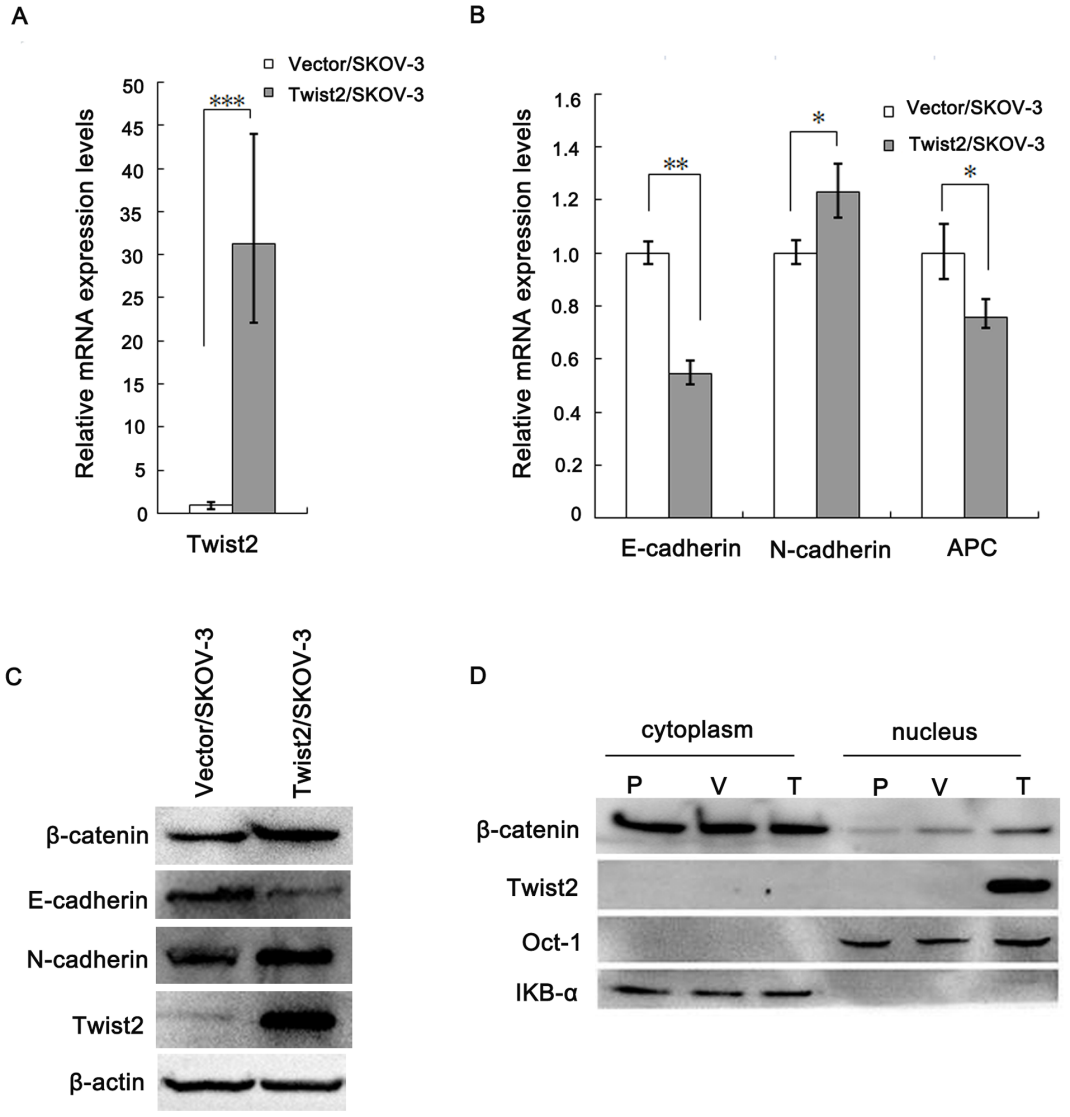
Twist2 changed the expressions of E-cadherin, N-cadherin, and distribution of β-catenin in SKOV-3 cells. **A and B**. Real time PCR results of Twist2, E-cadherin, N-cadherin, and APC mRNA expressing levels in the Twist2-expressing cells and the vector-transfected control cells (*P<0.05,**P<0.01,***P<0.001). **C.** Western Blot results of Twist2, E-cadherin, N-cadherin, β-catenin expression in the Twist2/SKOV-3 cells compared with the Vector/SKOV-3 control cells. **D.** Distribution of β-catenin in cytoplasm and nucleus detected by the cell fractionation method. P-parental SKOV-3 cancer cells, V-vector-transfected control cells, T-Twist2-expressing SKOV-3 cells.

EMT usually involves the disruption of tight junctions, adherens junctions, which contribute to the separation into individual cells [16], [17]. To further determine whether expression of Twist2 indeed promotes EMT in these ovarian cancer cells, we examined the expression of E-cadherin and N-cadherin, two well-established epithelial to mesenchymal makers, in Twist2-expressing and the vector-transfected SKOV-3 cells. We found that the expression of E-cadherin (epithelial maker) was remarkably reduced in Twist2-expressing cells relative to the control cells, but the expression of N-cadherin (mesenchymal maker) increased in the cells expressing Twist2 by real-time PCR (Fig. 5A, 5B). We further confirmed the expression results of E-cadherin, N-cadherin by Western Blot (Fig. 5C).

As Twist2/SKOV-3 cells displayed the morphological changes (Fig. 2B), enhanced potential to migrate (Fig. 4), and EMT markers expression, we concluded that ectopic expression of Twist2 promotes an EMT phenotype in epithelial ovarian carcinoma cells.

It is well recognized that the β-catenin–E-cadherin complex constitutes the structural core of the adherens contacts, and impedes β-catenin transfer to the nucleus and its transcriptional activity [18]. β-catenin was also up-regulated in the Twist2-expressing cells relative to the control cells with decreased E-cadherin (Fig. 5C). Figure 5D showed β-catenin distribution by cell fractionation method. Nuclear β-catenin increased in Twist2/SKOV-3 cells. In addition, APC mRNA decreased (Fig. 5B).

### 5. TOPflash transcriptional analysis of 293FT cells

**Figure 6:**
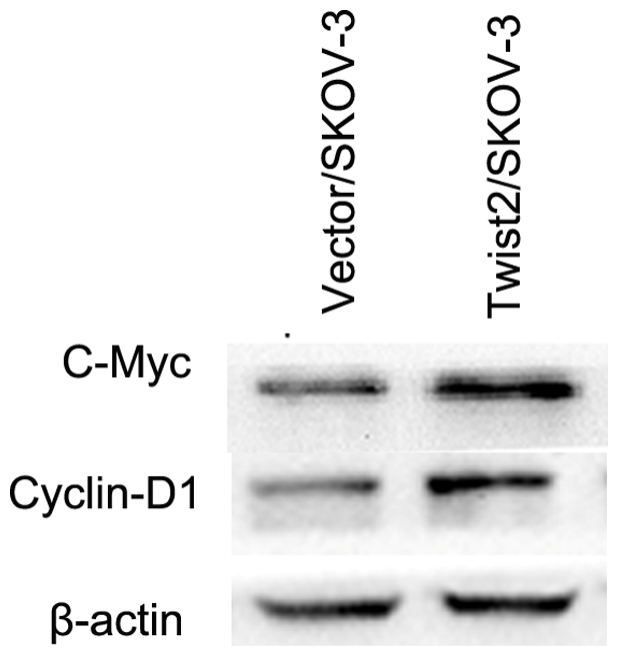
Downstream target proteins such as c-Myc, Cyclin-D1 in the Wnt signal pathway. As c-Myc and Cyclin-D1 are mostly regarded as Wnt target genes, we further examined their expressions by western blot repeated for three times. Immunoblot analysis showing that Both c-Myc and cyclin-D1 increased at the Twist2/SKOV-3 group compared with the Vector/SKOV-3 group in the representative figure.

**Figure 7:**
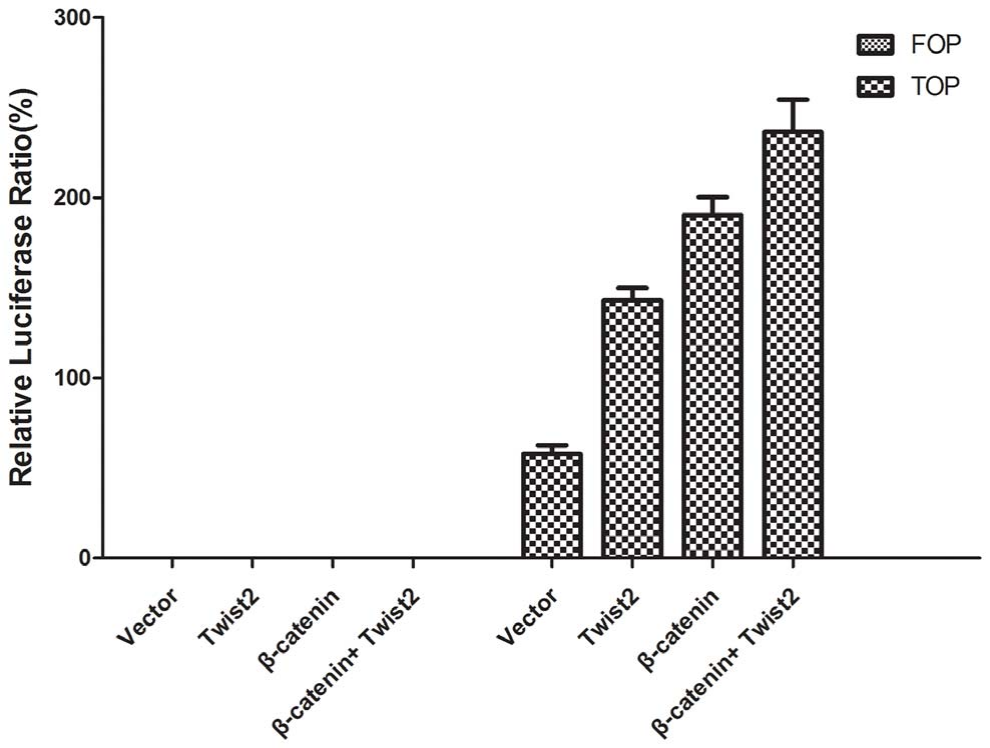
TCF/LEF-dependent transcriptional activity TOP/FOP flash. In order to measure TCF4/LEF1-dependent transcriptional activity of 293FT cell lines, cells were transfected with TOP/FOP flash reporter plasmids. After genes transfection for 48 h, transcriptional activity was measured by using Dual Luciferase Reporter Assay System. Cells in FOP flash groups showed negligible levels of transcriptional activity. In contrast, cells in TOPflash groups co-transfected with Twist2 showed significant increase in signal. The results are the average ± s.d. of three experiments (N = 3; P<0.001).

In order to detect the effect of Twist2-induced nuclear translocation of β-catenin on gene expression, we checked downstream target genes such as c-Myc, Cyclin-D1 in Wnt signal pathway. Both c-Myc and Cyclin-D1 were increased with Twist2 overexpression (Figure 6). We then measured TCF/LEF-dependent transcriptional activity using TOP/FOP flash reporter plasmids. After reporter genes were introduced into 293FT cells for 48 h, transcriptional activity was assessed by luciferase and renilla activity. 293FT cells showed significantly elevated LEF/TCF dependent transcription with Twist2 co-transfection (relative to vector control; N = 3; P<0.001; Figure 7).

Take together, these data demonstrate that Twist2 overexpression in SKOV-3 cells disrupts cell–cell adherens contacts and promotes cell migration. With the decreased E-cadherin, β-catenin released from E-cadherin/β-catenin complex. Meanwhile, β-catenin degradation reduced further accompanied by the decreased APC, which resulted in the accumulation of free β-catenin in the cytoplasm and caused β-catenin transfer to the nucleus to increase its transcriptional activity. These data indicated that nuclear β-catenin was involved in Twist2-induced EMT of ovarian cancer.

## Discussion

Ovarian cancer is a highly metastatic disease and the identification of molecules and/or signalling pathways involved in cancer invasion and metastasis is very important for which the early-stage detection is still a barrier [4]. Ovarian cancer cells are prone to metastasis throughout the peritoneal cavity mainly by intra-abdominal dissemination and by lymphatic dissemination. Metastasis is a complex process involving changes in extracellular matrix and cell–cell junctions. Epithelial-to-mesenchymal transition (EMT) is a necessary step towards metastatic tumor progression during detachment of tumor cells from the primary tumor site and attachment to metastatic sites. Few studies have examined the factors that promote EMT of epithelial ovarian cancer (EOC) cells [4], [19].

Several EMT-inducing regulators repress E-cadherin transcription, *via* interaction with specific E-boxes of the proximal E-cadherin promoter [20]. Most relevant are the Snail (SNAI1 and SLUG) [21], [22], ZEB (ZEB1 and ZEB2) [23] and basic helixloop-helix (bHLH) (Twist1 [24]) transcription factor families. Only recently, convincing evidence showed that Twist2 overexpression was significantly linked to cervical cancer progression [25], [26]. Twist2 activates EMT programs and promotes a cancer stem cell phenotype in breast cancer [10]. However, the biological function of Twist2 in tumor has remained mostly unknown.

In this study, we provide the first evidence that Twist2 expression is associated with high FIGO stage in ovarian cancer. It may be expected that Twist2 plays similar EMT functions in ovarian cancer. To this end, we introduced *twist2* into the SKOV-3 ovarian cancer cell line and examined cells biological behaviors including proliferation, migration and invasion. We found that over-expression of Twist2 had no effect on cell growth and Akt, Erk1/2 activation. Interesting, the Twist2/SKOV-3 cells increased the migration and invasion of ovarian cancer cells, as assessed by scratch-wound assay and invasion assay. As EMT results in enhanced cell motility and invasion, we further checked the EMT markers. A switch from E-cadherin to N-cadherin is also a key feature of EMT in ovarian cancer [4]. Overexpression of Twist2 significantly decreased E-cadherin and increased N-cadherin expression at both mRNA and protein levels respectively.

E-cadherin is a transmembrane glycoprotein of the type-I cadherin superfamily. Its cytoplasmic part is linked to the actin cytoskeleton via the catenins [27]. Canonical Wnt/β-catenin signaling has a major impact on EMT during cancer progression [28], [29]. Wnt signals inhibit GSK3β kinase activity to stop degradation and force a rapid increase in the levels of free, cytosolic β-catenin, and very soon thereafter, translocates into the nucleus where it binds to TCF/Lef transcription factors [30], [31]. A reduction in E-cadherin levels often releases β-catenin from adheren junction, resulting in nuclear β-catenin accumulation, and the consequent transactivation of a panel of target genes, among them those specifying several EMT-inducing factors [18], [28].

A recent published study on early cranial dermal development indicated a consistent role for Wnt/β-catenin signaling via Twist2 in dermal specification in different regions of the embryo [32]. Twist2 can be a direct transcription target and may mediate the functional role of Wnt signaling in dermal precursors. In our experiment, Twist2 increased nuclear β-catenin. Furthermore, soluble and nonsoluble cytosolic forms of β-catenin are strictly regulated by the proteosome/ubiquitination system that consists of GSK3β, axin, and adenomatous polyposis coli (APC) protein [25]. Due to downregulation of APC, β-catenin degradation was also repressed. Once in the nucleus, β-catenin would mediate the activation of specific mesenchymal genes. We showed an increase of c-Myc and Cyclin-D1 in Wnt/β-catenin signal pathway (Fig. 6). Because β-catenin was required for the expression of the mesenchymal genes, we checked whether the transcriptional activity of this protein was affected by ectopic Twist2. As shown in Fig. 7, the activity of the TCF4/LEF1-dependent promoter (TOP) was upregulated (two folds) in cells transfected with Twist2. Therefore, these results indicate that the expression of mesenchymal genes is under the control of β-catenin through TCF4/LEF1-dependent complexes. Confirmed by the result shown in Fig. 5D, nuclear β-catenin distribution was increased in Twist2/SKOV-3 cells. Thus, Twist2 might increase TCF4/LEF1-dependent β-catenin transcriptional activity.

In this study, we provide evidence that Twist2 affects cellular behaviors in SKOV-3 cells, including changes in morphology and motility. At the molecular level, Twist2 induces significant alteration of EMT markers. Furthermore, Twist2 promoted cell migration and invasion of SKOV-3 cells. Therefore, Twist2 promoted β-catenin release from E-cadherin/β-catenin complex through inhibition of E-cadherin. With nuclear β-catenin accumulation, Wnt/β-catenin pathway was then activated to promote an EMT phenotype.

To the best of our knowledge, we show for the first time that Wnt activation in Twist2-induced EMT progress in ovarian cancer, providing new insights into their metastasis.

## Conclusions

Our data suggest that upregulation of Twist2 is correlated with the FIGO stage in human ovarian cancers. Although there is no correlation between Twist2 expression and the tumor histological type, Twist2 is a potential indicator of high grade of malignancy and poor prognosis in clinical ovarian cancer. Overexpression of Twist2 induced the EMT phenotypes in human ovarian cancer cells *in vitro*. Downregulation of E-cadherin and APC as well as upregulation of N-cadherin and β-catenin (in cell nucleus), suggest that Twist2 promotes β-catenin release from E-cadherin/β-catenin through inhibition of E-cadherin. In addition, due to downregulation of APC, β-catenin degradation was repressed. Therefore, canonical Wnt/β-catenin pathway was then activated by nuclear β-catenin accumulation, thus evoked transcription of downstream target genes to promote tumor invasion and metastasis. Collectively, these data indicated that β-catenin is involved in Twist2-induced EMT in ovarian cancer.

## Materials and Methods

### Materials

Mouse anti-Twist2 antibody was purchased from Abnova Biotechnology. Mouse anti-Flag, anti-β-actin, and anti-Akt antibodies were purchased from Sigma Chemical Co. (St Louis, MO, USA). Rabbit anti-E-cadherin, Goat anti-N-cadherin, Rabbit anti-IKB-α, Rabbit anti-β-catenin, Mouse anti-Oct-1, Rabbit anti-Cyclin-D1, Rabbit anti-c-Myc, Mouse anti-rabbit IgG, anti-mouse IgG antibodies were purchased from Santa Cruz Biotechnology (Santa Cruz, CA, USA).

Rabbit anti-phospho-Akt (Ser 473) antibody was purchased from R&D Systems Europe. Rabbit anti-Erk1/2, Rabbit anti-p-Erk1/2, Rabbit anti-Akt, Rabbit anti-p-Akt were purchased from Cell Signaling Company. ABC Kits and DAB substrate kit were purchased from Thermo Scientific and Pierce, respectively. Formalin-fixed and paraffin-embedded ovarian cancer tissue microarray chips were from Alenabio Company. The research protocol and designs were approved by the Ethics Committee of Xiamen University (ID No: 20100602). All our clinical investigations were conducted according to the principles expressed in the Declaration of Helsinki.

### Immunohistochemical Staining

Tissue array sections in formalin-fixed and paraffin-embedded human primary ovarian cancer were examined to assess the expression of Twist2, and immunohistochemical staining (IHC) was performed as described previously [25], [33]. Twist2 was shown to specifically recognize the corresponding proteins (Fig. 1A). Diaminobenzidine was used to visualize the immunohistochemical reaction, followed by counterstaining with hematoxylin. To prepare the negative control, the primary antibody was replaced with normal mouse IgG. The IHC staining on tissue array was repeated for 3 times, analyzed through standard light microscopy. Positive cells showed brown granules in cytoplasm or cell nucleus.

The positive cell percentage was determined by calculating the percentage of positive cells in total observed cells: if <10%, 0;10%–20%, 1;20%–50%, 2;>50%, 3. The intensity was decided by comparing the staining of tumor cells: no staining or ambiguous staining, 0; weak staining, 1; medium staining, 2; strong staining, 3. The two scores were multiplied to categorize the staining: 0–1, negative (−);2–4, positive (+);≥5, strong positive (++).

### Generation of Twist2-overexpression Stable Ovarian Cancer Cells

The human ovarian cancer cell line SKOV-3 was obtained from ATCC. The cells were maintained in McCoy's 5A medium supplemented with 10% fetal bovine serum and penicillin/streptomycin. The Flag-Twist2-expressing plasmid had been constructed in our previous study [25]. The Flag-Twist2-expressing plasmid and the pBabe-puromycin vector were co-transfected into SKOV-3 ovarian cancer cells using the lipofectamine2000^TM^ transfection reagent (Invitrogen) according to the manufacturer's instruction. The Twist2-expressing stable clones and the vector control clones were obtained respectively through selection with puromycin. The Twist2 expression levels in the selected stable clones were then verified through immune-blot analysis with Twist2 and flag antibodies.

### Cell Morphological Observation and Confocal Immunofluorescent Staining

Cell morphological shapes were observed using phase microscopy (Olympus). Immunohistochemical staining was performed on Twist2/SKOV-3 and Vector/SKOV-3 ovarian cancer cells as previously described [25]. The staining of Twist2- and vector-transfected SKOV-3 cells were observed and analyzed using the laser scanning confocal microscopy (Olympus).

### Cell Growth Assay

The cells were seeded in normal medium until harvested. Cell growth was assessed via MTT assay as previously described [34]. The proliferation rate of the transfected cells was detected and compared with that of the vector control through viable cell counts using trypan-blue staining. Triplicate assays were performed in each group of cells. Data were expressed as mean ± SD. The cell proliferation rate (%) was calculated as follows: number of cells of the experimental group/number of the control group ×100%.

### Wound healing assay

Cells were seeded onto six-well plates (1×10^5^ cells/dish) and when they reached over 90% confluence, a scratch was made across the cell monolayer with the tip. Cells were gently washed with PBS for 3 times and maintained in the fresh medium. Cells were incubated for 24 hrs and photographed using an inverted tissue culture microscope at ×100 magnification. Assays were performed at least three times and data were presented as means ± SD. The migration potential between Twist2-expressing cells and the control cells was compared by relative gap distance. And we analyzed the results by the one way ANNOVA test (Prism5 software). A P-value of p<0.05 was considered statistically significant.

### Cell Invasion Assay

For invasion assay, 5×10^3^ cells were seeded on Matrigel-coated membrane inserts (BD Bioscience). The bottom chamber contained 0.75 ml McCoy's 5A supplemented with 10% fetal bovine serum as a chemoattractant. Cells were incubated for 24 hrs, the cells remaining inside the insert were removed with a cotton swab and cells that had penetrated the Matrigel to invade to the lower surface of the membrane were fixed in methanol and stained with H&E. After air drying the membrane, the cells were counted at ×100 magnification in 10 random fields of view under a microscope. Three independent experiments were performed in each case.

### Cell cycling analysis via Flow Cytometry

Cell cycling was identified via flow cytometry analysis as previously described [34]. Briefly, the cells were seeded for 24 h, then were harvested, washed twice with PBS, and fixed in 70% ethanol at 4°C overnight. The cell pellets were suspended in propidium iodide staining solution (20 μg/mL propidium iodide and 0.2 mg/mL RNase in PBS) and incubated for 30 min at 37°C. The samples were then analyzed through flow cytometry.

### Isolation of Subcellular Fractions, and Western Blot Analysis

Cell fractionation and Western blot analysis were performed as our previous publications [34], [35]. Details of cell fractionation is as following: Whole-cell extracts were prepared by scraping cells off Petri dishes, washing cell pellets twice in PBS, and then resuspending pellets in two-packed cell volumes of RIPA buffer [150 mM NaCl/50 mM Tris·HCl, pH 7.5/0.25% (wt/ol) deoxycholate/1% Nonidet P-40/5 mM sodium orthovanadate/2 mM sodium fluoride/protease inhibitor mixture]. Nuclear and cytoplasmic extracts were isolated by washing cell pellets in buffer A (10 mM Hepes, pH 7.5/10 mM KCl/1.5 mM Mg_2_Cl/0.5 mM NaF/1 mM glycerol phosphate/protease mixture), then lysis in buffer A+B (buffer A plus 0.5% Nonidet P-40) in a 2∶1 mix. After centrifugation (12,000×*g* for 10 min), supernatant was collected (cytoplasmic fraction). Pellets were washed in PBS, centrifuged (12,000×*g* for 10 min), and then flash-frozen in dry-ice–ethanol in buffer C (20 mM Hepes, pH, 7.5/420 mM NaCl/1.5 mM Mg_2_Cl/0.5 mM NaF/0.5 mM DTT/1 mM glycerol phosphate/protease mixture), followed by a slow thaw on ice. Pellets were then centrifuged (12,000×*g* for 10 min), washed, and membranes lysed in RIPA buffer. The supernatant was centrifuged (12,000×*g*) and collected (nuclear fraction). Protein Assay Kit for protein quantity analysis was purchased from Bio-Rad company (CA,USA). The enhanced chemiluminescence (ECL) detection system was purchased from Amersham (Amersham, IL, USA). All antibodies were described as materials. All the results are the average ± s.d. from at least three experiments.

### Real-time Reverse Transcriptase–PCR

Total cellular RNA was prepared using TRIzol reagent (Invitrogen) and the expression levels of E-cadherin, N-cadherin, APC, and Twist2 mRNAs were determined by real-time reverse transcriptase–PCR using SYBR Green [36]. Triplicate 10 ng aliquots of RNA were analyzed using two-step real time RT-PCR (Applied Biosystems), and relative mRNA levels determined using the <DELTA><DELTA>Ct method, using the GAPDH gene as the reference gene. All primer sets employed had amplification efficiencies ranging from 98.0+/−0.2% to 102.5+/−1.1% (R2 values for all reactions>or = 0.995). And non-template controls were used in duplicate per analysis. Data shown are normalized to GAPDH expression and represent the average of three repeated experiments. Primer sequences were as Table 2.

**Table 2:** Primers used for Real Time PCR.

Gene	Primer Forward	Primer Reverse
GAPDH	AGCCTCAAGATCATCAGCAATGCC	TGTGGTCATGAGTCCTTCCACGAT
Twist2	GCAAGAAGTCGAGCGAAGAT	GCTCTGCAGCTCCTCGAA
E-cadherin	AGAACAGCACGTACACAGCCCTAA	ATCAGCAGAAGTGTCCCTGTTCCA
N-cadherin	ACAGATGTGGACAGGATTGTGGGT	TATCCCGGCGTTTCATCCATACCA
APC	GAACAAGCATGAAACCGGCTCACA	TCCACCTTGGTTCCCAGATGACTT

### Transcriptional Reporter Gene Assay

TCF4/LEF1-dependent β-catenin transcriptional activity was assessed by using TOPflash and FOPflash reporter plasmid (the plasmids are provided by Professor Jianxing Ma). Briefly, the reporter genes were transfected into cells grown to semi-confluence in 12-well plates, and cultured for a further 48 h. Luciferase activity were measured by using Dual Luciferase Reporter Assay System (Promega, Madison, WI) according to the manufacturer's instructions. In order to see the effect of Twist2 on β-catenin, Twist2 plasmid was co-transfected into cells with TOPflash or FOPflash reporter genes, the luciferase and renilla activity was measured as described above.

### Statistical Analysis

The results of the experimental studies are expressed as mean ± SD. Statistical differences were analyzed through Student's t-test using SPSS 10.0 software; *p*<0.05 was considered to indicate statistical significance. Difference between groups was assessed by one way ANOVA tests (Prism5 software).

## References

[1] Khalil I, Brewer MA, Neyarapally T, Runowicz CD (2010) The potential of biologic network models in understanding the etiopathogenesis of ovarian cancer. Gynecol Oncol 116: 282–285. 19931138 10.1016/j.ygyno.2009.10.085

[2] Kobel M, Kalloger SE, Boyd N, McKinney S, Mehl E, et al (2008) Ovarian carcinoma subtypes are different diseases: implications for biomarker studies. PLoS Med 5: e232. 19053170 10.1371/journal.pmed.0050232 PMC2592352

[3] Ahmed N, Abubaker K, Findlay J, Quinn M (2010) Epithelial mesenchymal transition and cancer stem cell-like phenotypes facilitate chemoresistance in recurrent ovarian cancer. Curr Cancer Drug Targets 10: 268–278. 20370691 10.2174/156800910791190175

[4] Vergara D, Merlot B, Lucot JP, Collinet P, Vinatier D, et al (2010) Epithelial-mesenchymal transition in ovarian cancer. Cancer Lett 291: 59–66. 19880243 10.1016/j.canlet.2009.09.017

[5] Geiger TR, Peeper DS (2009) Metastasis mechanisms. Biochim Biophys Acta 1796: 293–308. 19683560 10.1016/j.bbcan.2009.07.006

[6] Zlobec I, Lugli A (2010) Epithelial mesenchymal transition and tumor budding in aggressive colorectal cancer: tumor budding as oncotarget. Oncotarget 1: 651–661. 21317460 10.18632/oncotarget.199 PMC3248128

[7] Haslehurst AM, Koti M, Dharsee M, Nuin P, Evans K, et al (2012) EMT transcription factors snail and slug directly contribute to cisplatin resistance in ovarian cancer. BMC Cancer 12: 91. 22429801 10.1186/1471-2407-12-91 PMC3342883

[8] Ye Y, Xiao Y, Wang W, Yearsley K, Gao JX, et al (2010) ERalpha signaling through slug regulates E-cadherin and EMT. Oncogene 29: 1451–1462. 20101232 10.1038/onc.2009.433

[9] Sanchez-Tillo E, Lazaro A, Torrent R, Cuatrecasas M, Vaquero EC, et al (2010) ZEB1 represses E-cadherin and induces an EMT by recruiting the SWI/SNF chromatin-remodeling protein BRG1. Oncogene 29: 3490–3500. 20418909 10.1038/onc.2010.102

[10] Fang X, Cai Y, Liu J, Wang Z, Wu Q, et al (2011) Twist2 contributes to breast cancer progression by promoting an epithelial-mesenchymal transition and cancer stem-like cell self-renewal. Oncogene 30: 4707–4720. 21602879 10.1038/onc.2011.181

[11] Li Y, Wang W, Yang R, Wang T, Su T, et al (2012) Correlation of TWIST2 up-regulation and epithelial-mesenchymal transition during tumorigenesis and progression of cervical carcinoma. Gynecol Oncol 124: 112–118. 22018873 10.1016/j.ygyno.2011.09.003

[12] Zhou C, Liu J, Tang Y, Zhu G, Zheng M, et al (2012) Coexpression of hypoxia-inducible factor-2alpha, TWIST2, and SIP1 may correlate with invasion and metastasis of salivary adenoid cystic carcinoma. J Oral Pathol Med 41: 424–431. 22103974 10.1111/j.1600-0714.2011.01114.x

[13] Liang X, Zheng M, Jiang J, Zhu G, Yang J, et al (2011) Hypoxia-inducible factor-1 alpha, in association with TWIST2 and SNIP1, is a critical prognostic factor in patients with tongue squamous cell carcinoma. Oral Oncol 47: 92–97. 21167768 10.1016/j.oraloncology.2010.11.014

[14] Gasparotto D, Polesel J, Marzotto A, Colladel R, Piccinin S, et al (2011) Overexpression of TWIST2 correlates with poor prognosis in head and neck squamous cell carcinomas. Oncotarget 2: 1165–1175. 22201613 10.18632/oncotarget.390 PMC3282075

[15] Hugo H, Ackland ML, Blick T, Lawrence MG, Clements JA, et al (2007) Epithelial–mesenchymal and mesenchymal–epithelial transitions in carcinoma progression. J Cell Physiol 213: 374–383. 17680632 10.1002/jcp.21223

[16] Giehl K, Menke A (2008) Microenvironmental regulation of E-cadherin-mediated adherens junctions. Front Biosci 13: 3975–3985. 18508491 10.2741/2985

[17] Chaw SY, Majeed AA, Dalley AJ, Chan A, Stein S, et al (2012) Epithelial to mesenchymal transition (EMT) biomarkers–E-cadherin, beta-catenin, APC and Vimentin–in oral squamous cell carcinogenesis and transformation. Oral Oncol 48: 997–1006. 22704062 10.1016/j.oraloncology.2012.05.011

[18] Orsulic S, Huber O, Aberle H, Arnold S, Kemler R (1999) E-cadherin binding prevents beta-catenin nuclear localization and beta-catenin/LEF-1-mediated transactivation. J Cell Sci 112 (Pt 8): 1237–1245. 10.1242/jcs.112.8.1237 10085258

[19] Pon YL, Zhou HY, Cheung AN, Ngan HY, Wong AS (2008) p70 S6 kinase promotes epithelial to mesenchymal transition through snail induction in ovarian cancer cells. Cancer Res 68: 6524–6532. 18701475 10.1158/0008-5472.CAN-07-6302

[20] Comijn J, Berx G, Vermassen P, Verschueren K, van Grunsven L, et al (2001) The two-handed E box binding zinc finger protein SIP1 downregulates E-cadherin and induces invasion. Mol Cell 7: 1267–1278. 11430829 10.1016/s1097-2765(01)00260-x

[21] Shimokawa M, Haraguchi M, Kobayashi W, Higashi Y, Matsushita S, et al.. (2012) The transcription factor Snail expressed in cutaneous squamous cell carcinoma induces epithelial-mesenchymal transition and down-regulates COX-2. Biochem Biophys Res Commun. 10.1016/j.bbrc.2012.12.035 23261444

[22] Miyoshi A, Kitajima Y, Sumi K, Sato K, Hagiwara A, et al (2004) Snail and SIP1 increase cancer invasion by upregulating MMP family in hepatocellular carcinoma cells. Br J Cancer 90: 1265–1273. 15026811 10.1038/sj.bjc.6601685 PMC2409652

[23] Huang W, Zhang Y, Varambally S, Chinnaiyan AM, Banerjee M, et al (2008) Inhibition of CCN6 (Wnt-1-induced signaling protein 3) down-regulates E-cadherin in the breast epithelium through induction of snail and ZEB1. Am J Pathol 172: 893–904. 18321996 10.2353/ajpath.2008.070899 PMC2276413

[24] Vesuna F, van Diest P, Chen JH, Raman V (2008) Twist is a transcriptional repressor of E-cadherin gene expression in breast cancer. Biochem Biophys Res Commun 367: 235–241. 18062917 10.1016/j.bbrc.2007.11.151 PMC2696127

[25] Mao Y, Zhang N, Xu J, Ding Z, Zong R, et al (2012) Significance of heterogeneous Twist2 expression in human breast cancers. PLoS One 7: e48178. 23133563 10.1371/journal.pone.0048178 PMC3485060

[26] Mao Y, Xu J, Song G, Zhang N, Yin H (2013) Twist2 promotes ovarian cancer cell survival through activation of Akt. Oncol Lett 6: 169–174. 23946798 10.3892/ol.2013.1316 PMC3742652

[27] Hsu YM, Chen YF, Chou CY, Tang MJ, Chen JH, et al (2007) KCl cotransporter-3 down-regulates E-cadherin/beta-catenin complex to promote epithelial-mesenchymal transition. Cancer Res 67: 11064–11073. 18006853 10.1158/0008-5472.CAN-07-2443

[28] Schmalhofer O, Brabletz S, Brabletz T (2009) E-cadherin, beta-catenin, and ZEB1 in malignant progression of cancer. Cancer Metastasis Rev 28: 151–166. 19153669 10.1007/s10555-008-9179-y

[29] Beiter K, Hiendlmeyer E, Brabletz T, Hlubek F, Haynl A, et al (2005) beta-Catenin regulates the expression of tenascin-C in human colorectal tumors. Oncogene 24: 8200–8204. 16091738 10.1038/sj.onc.1208960

[30] MacDonald BT, Tamai K, He X (2009) Wnt/beta-catenin signaling: components, mechanisms, and diseases. Dev Cell 17: 9–26. 19619488 10.1016/j.devcel.2009.06.016 PMC2861485

[31] Gordon MD, Nusse R (2006) Wnt signaling: multiple pathways, multiple receptors, and multiple transcription factors. J Biol Chem 281: 22429–22433. 16793760 10.1074/jbc.R600015200

[32] Tran TH, Jarrell A, Zentner GE, Welsh A, Brownell I, et al (2010) Role of canonical Wnt signaling/ss-catenin via Dermo1 in cranial dermal cell development. Development 137: 3973–3984. 20980404 10.1242/dev.056473 PMC2976281

[33] Song G, Ouyang G, Mao Y, Ming Y, Bao S, et al (2009) Osteopontin promotes gastric cancer metastasis by augmenting cell survival and invasion through Akt-mediated HIF-1alpha up-regulation and MMP9 activation. J Cell Mol Med 13: 1706–1718. 19602039 10.1111/j.1582-4934.2008.00540.x PMC6512381

[34] Mao Y, Song G, Cai Q, Liu M, Luo H, et al (2006) Hydrogen peroxide-induced apoptosis in human gastric carcinoma MGC803 cells. Cell Biol Int 30: 332–337. 16533611 10.1016/j.cellbi.2005.12.008

[35] Xia W, Liu Z, Zong R, Liu L, Zhao S, et al (2011) Truncated ErbB2 Expressed in Tumor Cell Nuclei Contributes to Acquired Therapeutic Resistance to ErbB2 Kinase Inhibitors. Mol Cancer Ther 10: 1367–1374. 21673090 10.1158/1535-7163.MCT-10-0991 PMC3836594

[36] Bustin SA, Benes V, Garson JA, Hellemans J, Huggett J, et al (2009) The MIQE guidelines: minimum information for publication of quantitative real-time PCR experiments. Clin Chem 55: 611–622. 19246619 10.1373/clinchem.2008.112797

